# Determination of Five Phthalate Esters in Tea and Their Dynamic Characteristics during Black Tea Processing

**DOI:** 10.3390/foods11091266

**Published:** 2022-04-27

**Authors:** Yanyan Tang, Mengxin Wang, Cheng Pan, Shuishan Mi, Baoyu Han

**Affiliations:** Zhejiang Provincial Key Laboratory of Biometrology and Inspection and Quarantine, College of Life Sciences, China Jiliang University, Hangzhou 310018, China; yan9268264@163.com (Y.T.); wmx@cjlu.edu.cn (M.W.); pancheng@cjlu.edu.cn (C.P.); mishuishan09@163.com (S.M.)

**Keywords:** black tea processing, phthalate esters, solid-phase extraction, processing factor, octanol–water partition coefficient

## Abstract

A highly specific and high extraction-rate method for the analysis of dimethyl phthalate (DMP), diethyl phthalate (DEP), di-n-butyl phthalate (DBP), diisobutyl phthalate (DiBP), and di-(2-ethyl) hexyl phthalate (DEHP) in tea samples was developed. Based on three-factor Box–Behnken response surface design, solid-phase extraction (SPE) of five phthalate ester (PAE) residues in tea was optimized. Optimal extraction conditions were found for extraction temperature (40 °C), extraction time (12 h), and ratio of tea to n-hexane (1:20). The dynamic distribution of PAEs at each stage of black tea processing was also analyzed, and it was found that the baking process was the main stage of PAE emission, indicating that traditional processing of black tea significantly degrades PAEs. Further, principal component analysis of the physicochemical properties and processing factors of the five PAEs identified the main processing stages affecting the release of PAEs, and it was found that the degradation of PAEs during black tea processing is also related to its own physicochemical properties, especially the octanol–water partition coefficient. These results can provide important references for the detection, determination of processing losses, and control of maximum residue limits (MRLs) of PAEs to ensure the quality and safety of black tea.

## 1. Introduction

Phthalate esters (PAEs) are a type of additives used to enhance the flexibility of materials; they are widely used in plastic products and account for 80% of the total plasticizers [1,2]. PAEs are not chemically bonded to the polymer matrix, and therefore, they are easily released from the plastic and enter the environment, where they are difficult to degrade [3,4]. It is well known that PAEs can interfere with the human endocrine system and have teratogenic, carcinogenic, and mutagenic potential [5,6,7,8]. The United States Environmental Protection Agency has listed six PAEs, including dimethyl phthalate (DMP), diethyl phthalate (DEP), di-n-butyl phthalate (DBP), butyl benzyl phthalate (BBP), di-(2-ethyl) hexyl phthalate (DEHP), and di-n-octyl phthalate (DnOP), as toxic pollutants for priority control [9]. China has also issued a number of relevant standards and regulations to regulate PAEs, especially to control the contamination of food by PAEs. At this point, PAEs have become widespread environmental pollutants, and there are a lot of reports of PAEs in food [10,11] and materials in contact with food [12,13], with a focus on sample pretreatment and detection methods. Pretreatment methods mainly include solid-phase extraction (SPE) [14], solid-phase microextraction (SPME) [15], liquid–liquid extraction (LLE) [16], dispersive liquid–liquid microextraction (DLLME) [17], gas–liquid micro-extraction (GLME) [18], microwave-assisted extraction (MAE) [8,19], and isotope dilution [20]. The methods to detect PAEs mainly include supercritical fluid chromatography [21], gas chromatography–mass spectrometry (GC–MS) [22], gas chromatography tandem mass spectrometry (GC–MS/MS) [10,23], high performance liquid chromatography (HPLC) [24], high-performance liquid chromatography–electrospray ionization-mass spectrometry (HPLC–ESI-MS) [25], and liquid chromatography tandem mass spectrometry (LC-MS/MS) [26,27]. It can be seen that many scholars have been paying attention to and studying PAEs pollutants, however, there are few studies on the content and pollution characteristics of PAEs in teas.

In the 1970s to 1980s, domestic researchers in China occasionally detected residual PAEs from green tea and black tea. In the past 20 years, more and more studies have reported the detection of PAEs in various types of tea. Now, the probability of detecting PAEs in fresh tea leaves, tea during processing, and finished tea is 100%, which indicates all aspects of tea production are likely contaminated by PAEs. PAEs include more than 20 compounds, of which DBP, di-isobutyl phthalate (DiBP), DEP, DEHP, and DMP are the most commonly detected in tea [28,29]. Several methods of detecting PAEs in tea and tea infusions have been established [25,26,28,29,30], and the total loss rate of PAEs during green tea processing has also been investigated; for example, the loss rate of PAEs caused by the drying process accounts for 61.3–73.7% of the total loss rate [29].

Black tea makes up the highest proportion of tea production and sales in the world, accounting for approximately 70% of the total tea products globally. Black tea originated from China hundreds of years ago and gradually evolved into three types: congou black tea, souchong black tea, and broken black tea. Usually, countries other than China only produce broken black tea. Frequently, the detection of PAEs in black tea has aroused the concern of the tea industry. However, the dynamic characteristics of PAE residues in black tea processing, methods to prevent PAEs from contaminating black tea, and means of removing a portion of PAEs effectively by processing remain unclear.

Congou black tea is mainly produced in China and is the major type of black tea. It is processed from fresh leaves, usually through four processes: withering, rolling, fermentation, and drying (primary drying and baking). In this study, we developed a detection method to enrich, purify, and extract the PAEs in tea using solid-phase extraction technology. GC–MS was used for the qualitative and quantitative analysis of the extracted PAEs. Later, we used the established method to detect the content and changes of DMP, DEP, DiBP, DBP, and DEHP in each stage of black tea production, and evaluated their dynamic characteristics and possible causes. We expect to develop an environmentally friendly and highly efficient method for the extraction and accurate quantification of PAEs in tea, and to use the method to determine and evaluate the residual dynamics, variation, and pollution levels of PAEs during black tea processing. This method can provide a reference for the prevention and suppression of the pollution of black tea by PAEs.

## 2. Materials and Methods

### 2.1. Reagents and Standards for PAEs

Five PAE analytical standards (≥99.0%) of DMP, DEP, DiBP, DBP, and DEHP were purchased from Dr. Ehrenstorfer GmbH (Augsburg, Germany). Optima GC–MS grade toluene, acetonitrile, and n-hexane were purchased from Tedia Co., Inc. (Fairfield, OH, USA). Analytical grade sodium chloride and anhydrous ethanol were purchased from Sinopharm Chemical Reagent Co., Ltd. (Shanghai, China). Solid-phase extraction glass columns (CARB/NH_2_, 1000 mg/6 mL) were purchased from Dikma Technologies Inc. (Beijing, China).

To remove possible cross-contamination of PAEs, all glassware and chinaware used in the study were immersed in methanol overnight, rinsed with hexane, and dried at 140 °C for at least 4 h. Organic solvents were redistilled before being used. Sodium chloride was heated at 450 °C for 4 h in a muffle furnace and kept in sealed glass vials after cooling. For PAE analysis, a procedural blank and solvent blank were run with every batch of samples for quality assurance and quality control.

### 2.2. Tea Sampling

In April 2021, fresh and tender tea shoots were plucked and processed to black tea using the following process in an organic tea garden in Zhejiang Gengxiang Organic Tea Development Co., Ltd., Wuyi County, Zhejiang, China. The tea variety Jiukeng, aged more than 6 years, was chosen for the present study. The standard for fresh tea shoots is one bud and two leaves. One bud and two leaves were transported to the tea processing workshop, spread to dry for 0.5 h at 25 °C, withered for 6 h at 32 °C, rolled for 1.5 h at 25–28 °C, fermented for 3 h at 35 °C, primary dried for 8 min at 110 °C, and far-infrared aroma-enhanced dried (far-infrared aroma- enhanced machine model: DXCWS-05, Jiangsu, China) for 14 min at 100 °C before storage in glass bottles for tea sampling.

The sampling process proceeded according to national standard GB/T 8302-2013 [31]. In short, sampling was conducted at six stages during tea processing: plucking of fresh leaves, end of withering (withered sample), end of rolling (rolled sample), end of fermentation (fermented sample), end of initial drying (first drying sample), and end of far-infrared aroma-enhanced baking sample (finished tea sample). Samples were immediately stabilized in liquid nitrogen and stored in a −80 °C ultra-low temperature freezer for later usage.

### 2.3. Construction of Standard Curves of PAEs

Mixed standard stock solution was prepared. Briefly, equal amounts of DMP, DEP, DiBP, DBP, and DEHP were pipetted into a 100 mL volumetric flask and accurately diluted with n-hexane to a final concentration of 1 μg/mL mixed standard stock solution. Mixed standard stock solutions were stored at −20 °C until further usage.

Matrix-matched standard working solution was prepared. Briefly, the above mixed standard stock solution was serially diluted with the blank sample. The matrix-matched standard working solution was made fresh with concentrations of 0 μg/L, 1 μg/L, 5 μg/L, 10 μg/L, 20 μg/L, 50 μg/L, 100 μg/L, 200 μg/L, 500 μg/L, and 1000 μg/L; these were used to make the standard working curve (Table S1).

### 2.4. Extraction of PAEs in Tea

The method used to determine PAEs in tea was optimized and modified from the national standard GB 5009.271-2016 [32]. In short, 2 g of crushed and mixed tea samples were accurately weighed and put into a 40 mL glass centrifuge tube. Then, 2 g of sodium chloride and 20 mL of n-hexane were added. The samples were then mixed using a vortex shaker for 1 min. Samples were placed in a 40 °C water bath for 12 h, then were centrifuged at 3000 r min^−1^ for 2 min at 10 °C (Eppendorf, Mississauga, ON, Canada) for collection of the supernatant. Another 20 mL n-hexane was added to the precipitate, which was then shaken and centrifuged at 3000 r/min for 2 min at 10 °C. The supernatants were collected and combined with the previous supernatant. The supernatants were distilled to near dry by rotary evaporation in a 40 °C water bath (Rikakikai Co., LTD, Tokyo, Japan). Then, 8 mL of acetonitrile–toluene (3:1, *v*/*v*) mixture was added to dissolve the dried supernatant, which was then transferred into a glass SPE column, and the effluent was collected. The glass SPE column was activated with 10 mL of acetonitrile–toluene (3:1, *v*/*v*) mixture, and the effluent was discarded before use. Another 22 mL of acetonitrile–toluene (3:1, *v*/*v*) mixture was added to the column. The second eluate was collected and combined with the previous one. The eluates were distilled by rotary evaporation in a 40 °C water bath to dry. The final extracts were cooled and reconstituted with 1 mL n-hexane, vortexed, and filtered (0.45 μm cellulose acetate syring filters) for GC–MS analysis.

### 2.5. Optimization of PAE Extractions in Tea Using Response Surface Methodology

The response surface method is a method to optimize processing workflows and experimental conditions and is suitable for solving nonlinear data processing problems [33]. Three factors, including solid to liquid ratios (1:10, 1:15, 1:20, 1:25, 1:30), extraction time (3 h, 6 h, 9 h, 12 h, 18 h, 24 h), and extraction temperature (20 °C, 30 °C, 40 °C, 50 °C, 60 °C), were set up and tuned to optimize the above extraction conditions by using the response surface method. Single-factor experiments were performed first, and each experiment was conducted in triplicate.

Later, based on the results from the single-factor test, the solid-to-liquid ratio (A), extraction temperature (B), and extraction time (C) were used as the independent variables of the response surface test. The amount of extracted PAEs in tea (Y) was used as the response value, and the process was optimized by Box–Behnken analysis. Variance analysis and response contour map analysis were carried out to optimize the extraction conditions of PAEs in tea. The optimal conditions were used to predict the response in other experiments.

### 2.6. GC–MS Analysis

Samples were analyzed by gas chromatography–mass spectrometry (GC–MS) using an Agilent 6890A GC coupled to an MSD 6975 (Agilent Technologies, Wilmington, DE, USA) with an HP-5MS quartz capillary column (30.0 m × 250 μm × 0.25 μm). The injector volume was 1 μL in splitless mode, and the inlet temperature was set to 300 °C. The flowrate of the carrier gas (99.999% high-purity helium) was 1.0 mL min^−1^. The temperature was programmed as follows: the initial temperature of 80 °C was held for 1 min; the temperature was raised to 140 °C at 5 °C/min and held for 2 min; the temperature was raised to 170 °C at 3 °C/min and held for 2 min; then the temperature was increased to 280 °C at 5 °C/min and held at 280 °C for 1 min. The solvent delay was 5 min. The mass spectrometry was set up as follows: EI source temperature, 230 °C; ionization energy, 70 eV; and quadrupole temperature, 150 °C. Full scan mode was used for the qualitative analysis, with a mass range of 30–400 *m*/*z*.

### 2.7. Method Validation

The accuracy of the developed extraction method was validated by evaluating the linearity, accuracy, precision, limit of quantification, standard recovery, and matrix effect. The matrix effect (ME) was evaluated by analyzing the ratio of the slope of the matrix-matched standard curve to the slope of the solvent standard curve [34]. If the ME is higher than 1, the matrix has an enhancing effect on the result. On the other hand, an ME < 1 indicates an inhibitory effect. According to the linear range of the five PAEs and the dilution ratio of the sample pretreatment, the mixed standard solutions with three concentration gradients (100 μg/mL, 500 μg/mL, and 1000 μg/mL) were added to the blank tea samples. Each gradient contained three parallel samples. PAE extraction was carried out under the optimum extraction conditions according to the method described. The recoveries and the relative standard deviation of five PAEs were also calculated.

### 2.8. Statistical Data Analysis

One-way analysis of variance (ANOVA) was performed using SPSS 22.0, and multiple comparison analysis was performed using Duncan’s new multiple range test. Design-Expert V8.0.6.1 (Stat-Ease, Inc., Minneapolis, MN, USA) was used for the experimental design of Box–Behnken central combined experiment and data processing [35].

The processing factor (PF) proposed by the United States Environmental Protection Agency (EPA) was used to evaluate the effect of processing technology on the content of PAEs in tea during processing. PF > 1 indicates a processing enrichment effect, and PF < 1 suggested a processing dilution effect [36]. PF was calculated as follows:

PF = M_2_/M_1_ (1)

where M_1_ is the level of PAE residues in fresh tea (μg/kg), and M_2_ is the level of PAE residues after processing (μg/kg).

## 3. Results

### 3.1. Effects of Single Factors on Extraction of PAEs from Tea

**Figure 1:**
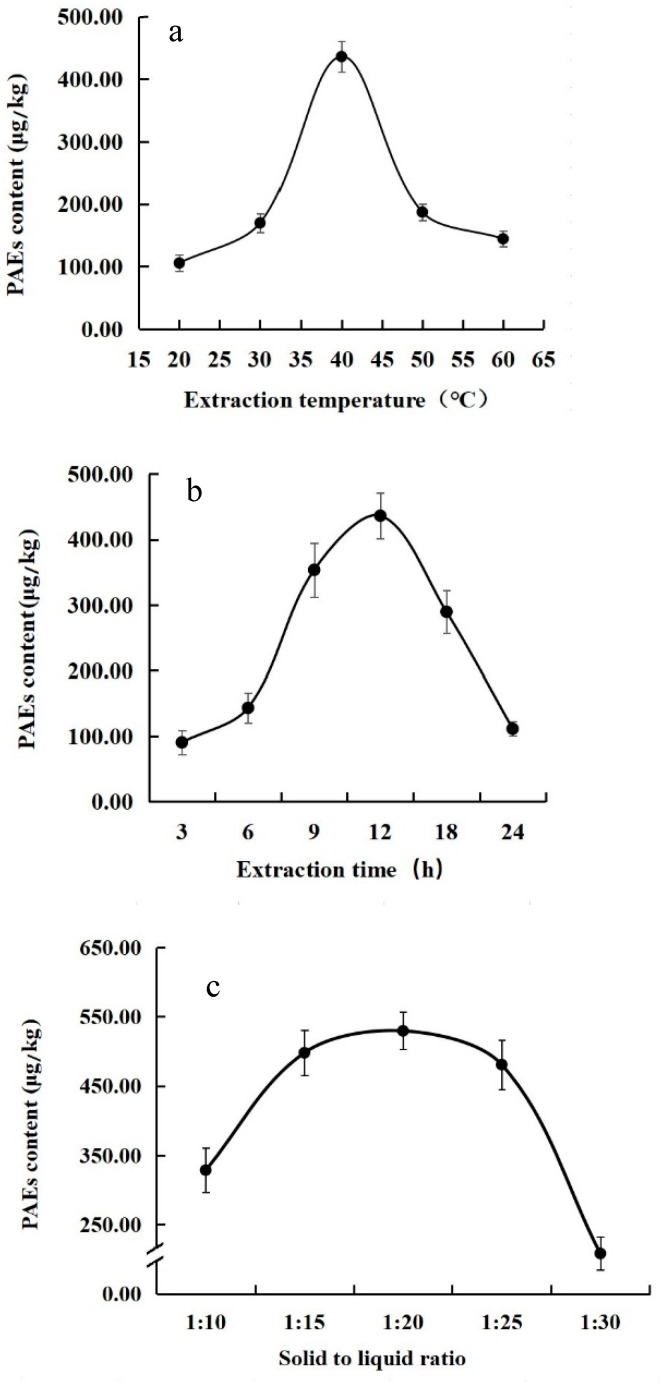
Effect of extraction temperature (**a**), extraction time (**b**), and solid–liquid ratio (**c**) on the extraction of PAEs from tea.

Single-factor experiments were carried out with different extraction temperatures as the single variable. The extraction conditions were as follows: fresh leaves of Jiukeng cultivar 2 g, solid to liquid ratio 1:20, extraction time 12 h, and different temperatures of 20 °C, 30 °C, 40 °C, 50 °C, and 60 °C. As the extraction temperature increased, the amount of extracted PAEs from tea first increased and then decreased, reaching peak extraction at a temperature of 40 °C (Figure 1a). Among the five PAEs detected, DBP accounted for the largest proportion (57.40–87.85%), DEHP accounted for the smallest proportion (2.25–9.29%), and DMP, DEP, and DiBP showed little difference.

Single-factor experiments were carried out with different extraction times as the single variable. The extraction conditions were as follows: fresh leaves of Jiukeng cultivar 2 g, solid–liquid ratio 1:20, extraction temperature 40 °C, and different extraction times were set as 3 h, 6 h, 9 h, 12 h, 18 h, and 24 h. It was found that with the increase of extraction time, the amount of extracted PAEs in tea also first increased and then decreased, and the extraction amount was the highest when the extraction time was 12 h (Figure 1b). Similarly, DBP accounted for the highest proportion (31.45–87.24%) of the five PAEs extracted at the different extraction times, and the amount of extracted DBP changed most significantly with the extension of extraction time. The extraction amount of the other four PAEs did not change significantly within the different extraction times.

Single-factor experiments were carried out with different solid–liquid ratios as the single variable. The extraction conditions were as follows: fresh leaves of Jiukeng cultivar 2 g, extraction temperature 40 °C, extraction time 12 h, and different solid–liquid ratios of 1:10, 1:15, 1:20, 1:25, and 1:30. The results suggest that the amount of PAEs extracted from tea was the highest with a solid-to-liquid ratio of 1:20. The amount of DBP accounted for the largest proportion (56.17–66.97%) of the total content of PAEs, and DEHP content was the least (4.45–6.27%) (Figure 1c). No significant differences were found among the amount of DMP, DEP, and DIBP.

### 3.2. Response Surface Optimization Experiment

Based on the results from single-factor experiments and the principle of central combination design of the Box–Behnken response surface method [33], a three-factor and three-level response surface experiment was designed. This experiment focused on identifying the effect of each factor on the extraction of PAEs in tea and the interaction between the factors. The solid–liquid ratio (A), extraction temperature (B), and extraction time (C) were chosen as three independent variables, and the amount of PAEs extracted from tea was used as the dependent variable (Y, response value). All other conditions were the same. The detailed experimental design and results of the response surface experiment are shown in Table 1, including the experimental values and those predicted by the model. The following multiple regression equation, obtained by Design-Expert V8.0.6.1 software, using multiple regression fitting of the data in Table 1 is shown: Y = 567.92 − 17.28A + 28.44B + 107.34C − 10.93AB − 8.77AC + 4.24BC − 158.79A^2^ − 137.54B^2^ − 140.13 C^2^.

The experimental data were analyzed by ANOVA, and the significance of the coefficients of the regression was evaluated by their corresponding *p*-values. As Table 2 indicates, the *p*-value of the model (*p* < 0.01) indicates that the model is significant. The lack-of-fit *p*-value of 0.4980 implies that it was not significant (no lack-of-fit factor observed). These values indicate that the regression equation can be used to analyze the experimental results. The adjusted coefficient of determination of the model (*R*^2^*_adj_* = 0.9202) was very high and indicated that the model had only 7.98% of the variation that can be explained by the model. The following parameters in the equation indicate that the model is significant, the degree of fit was high, the error of experimental prediction was small, and the data were reproducible: correlation coefficient *R*^2^ = 0.9651, predicted *R*^2^ = 0.7364, the difference between *R*^2^ and *R*^2^*_adj_* < 0.1, the difference between *R*^2^*_adj_* and predicted *R*^2^ < 0.2, Adeq Precision = 11.872 > 4. These results suggest the equation has good performance and reflects the effect of three factors on the extraction of PAEs from tea. Factors C, A^2^, B^2^, and C^2^ all showed a highly significant effect (*p* < 0.05). The order of the influence of the three factors on the extraction of PAEs was as follows: C > B > A.

**Figure 2:**
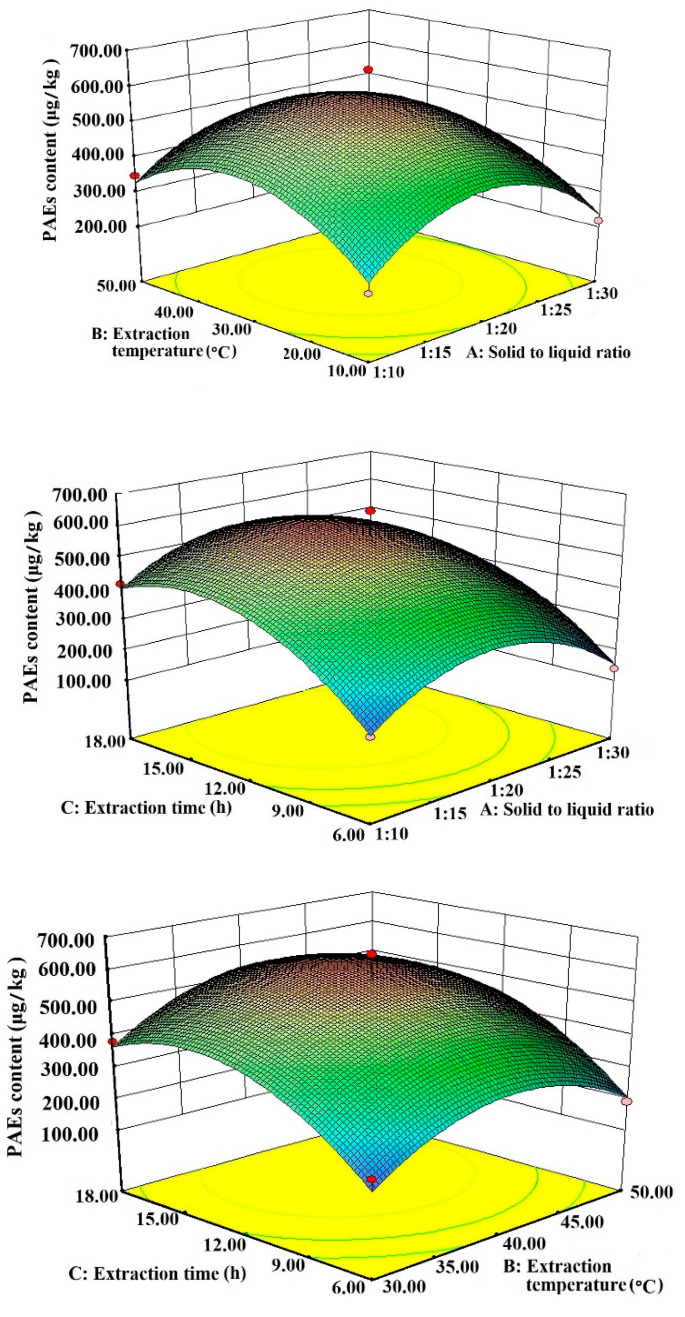
Response surface map for interaction of various factors.

The 3D response surface map of the interaction of solid–liquid ratio, extraction time, and extraction temperature on the extraction of tea PAEs is shown in Figure 2. The response surface map is a three-dimensional surface map formed by the extracted amount of PAEs from tea and various influencing factors, and reflects the interaction between the optimal extraction points and different parameters. If the curve is steeper, the effect of this factor on the extraction of PAEs from tea is greater. Extraction time has the greatest influence on the extraction of PAEs, followed by extraction temperature, and the solid-to-liquid ratio has the least influence (Figure 2). The interaction between the solid-to-liquid ratio and extraction temperature is the largest, followed by the solid-to-liquid ratio and extraction time, and the extraction temperature and extraction time shows the least obvious interaction.

According to the results of response surface analysis, the optimal conditions for the extraction of PAEs from tea are: solid-to-liquid ratio 1:20, extraction temperature 40 °C, and extraction time 12 h. Under these conditions, the theoretical prediction of PAE content is 585.85 μg/kg. For validation of the optimal extraction conditions, three groups of parallel experiments were performed. The average measured and the relative error were 574.50 μg/kg and 1.98%, respectively, which suggests that the prediction of the regression model is accurate, reliable, and reproducible.

### 3.3. Validation of the Developed Method

**Figure 3:**
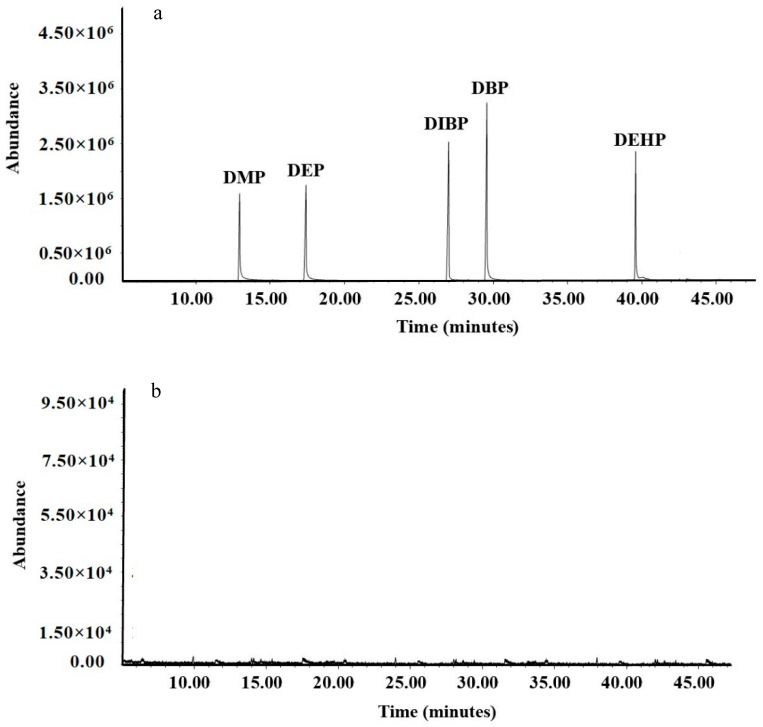
Total ion chromatogram of five PAEs; standard (**a**) and n-hexane solution (**b**).

A matrix-matched working standard solution with a concentration in the range of 1–1000 μg/L was prepared. The standards were measured under the predetermined GC–MS conditions, and a calibration curve was established. The total ion chromatograms of the five PAEs and n-hexane solution are shown in Figure 3, and their mass spectrometry characteristics are shown in Table 3. The five compounds all had good discrimination and flat baseline, and the noise in the chromatogram can be ignored. These results suggest that the detection of PAEs using GC–MS is reliable.

The linearity, accuracy, precision, the limit of detection (LOD), and limit of quantification (LOQ) of PAEs were performed (Table 4). Linearity was obtained at *r*^2^ > 0.99 for PAEs ranging from 1 to 1000 μg/L (except for 500 μg/L for DBP). LOD and LOQ were calculated according to the method of GB/T 27417-2017 [37] by the detection of actual samples and low-concentration spiked samples. Using 3- and 10-fold signal-to-noise ratios (S/N), the LODs of the five PAEs were 0.2–0.6 μg/kg, and the LOQs were 0.7–2.0 μg/kg.

The accuracy of the method was verified by measuring the recovery of spiked PAEs with standard solutions at three levels: 100, 500, and 1000 μg/L for tea. Three parallel samples were prepared for each gradient, and the results are shown in Table 4. The recoveries of the five PAEs ranged from 80.70% to 98.68%, and the relative standard deviations (*n* = 6) of the five PAEs were from 2.7% to 8.1% (all less than 10%). The matrix effects of the five PAEs in the blank matrix were investigated at five different levels: 20, 50, 100, 200, and 500 μg/L. The results show that the matrix effects of the five PAEs compounds are 0.4–1.1, which suggests the interference of the matrix on the compounds was small. 

### 3.4. Dynamic Changes of PAEs during Black Tea Processing

In this study, a total of six batches of samples at different stages during black tea processing were tested three times using the same apparatus. After the samples from each processing stage were extracted and analyzed according to the above method, the content of five PAEs in the samples was quantified using external standards (Table 5). In order to compare and analyze the change of PAE levels during black tea processing, the content of PAEs at each stage was converted into dry matter.

**Figure 4:**
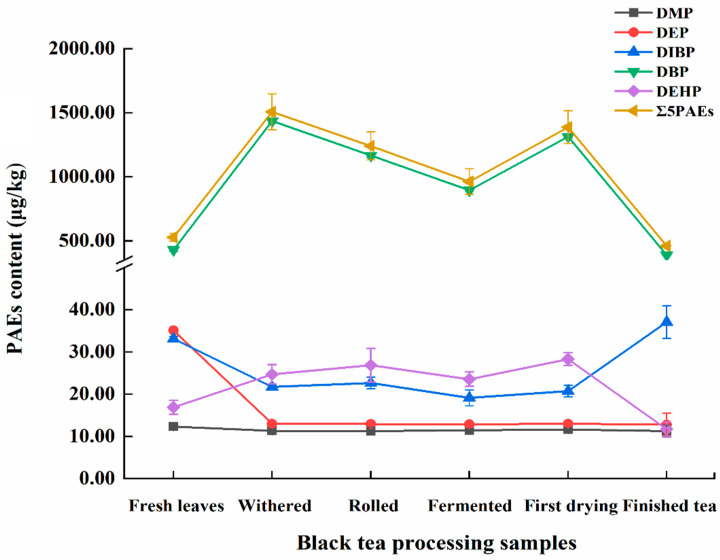
PAE content (±SD (μg/kg)) at each stage of black tea processing.

As shown in Figure 4, five PAEs (DMP, DEP, DiBP, DBP, and DEHP) were all detected during black tea processing, among which the content of DBP was the highest (81.5–95.30% of the total PAEs) and DMP was the lowest. Consistent with the trend of DEHP, the amount of DBP showed a trend of increasing first and then decreasing during the whole process. During the whole process, the content of DBP and DEHP increased significantly in withering, rolling, fermentation, and initial drying, while their content decreased sharply after far-infrared baking, with a significant loss rate of 70.49% and 58.60%, respectively. The contents of DMP, DEP, and DiBP were all significantly reduced in each stage of black tea processing. Compared with fresh leaves, the loss rates of DMP and DEP after far-infrared baking were 8.53% and 63.50%, respectively.

In each stage of black tea processing except for the finished tea sample, the total content of PAEs was generally higher than that in fresh leaves, especially in the withered sample (up to 1507.21 μg/kg), followed by the initial drying sample with a content of PAEs of 1387.61 μg/kg. However, after far-infrared baking, the content of PAEs dropped sharply, with the loss rate up to 66.79%. In addition to far-infrared baking, the content of PAEs during rolling and fermentation also decreased to a certain extent, with loss rates of 17.67% and 22.39%, respectively.

**Figure 5:**
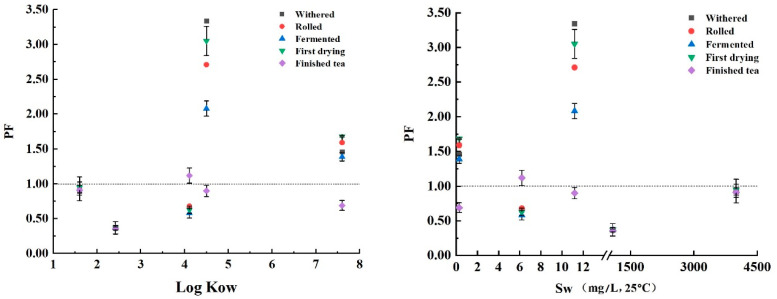

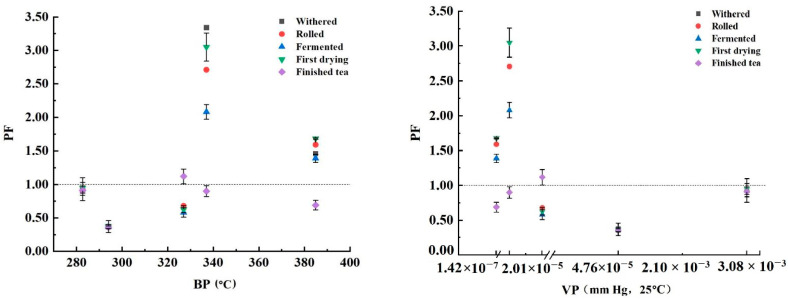
Effects of physicochemical properties of five PAEs on processing factors during black tea processing.

The processing factor (PF) was used to evaluate the change of PAEs during tea processing and can effectively indicate the main factors causing PAE degradation. The PFs during withering, rolling, fermentation, initial drying, and far-infrared baking were 2.86, 2.35, 1.82, 2.63, and 0.87, respectively, which suggests far-infrared baking is the main cause of the degradation of PAEs during black tea processing. In each stage of black tea processing, the effects of physicochemical properties of five PAEs on the PFs were analyzed. The physicochemical properties of five PAEs are shown in Table 6. Figure 5 shows an increasing trend with the increase of the octanol–water partition coefficient (Log Kow) and boiling point (Bp). Specifically, when Log Kow was less than 4 and Bp was less than 300 °C, the PFs were less than 1, which indicates low residue levels of PAEs. On the contrary, the PFs showed an overall decreasing trend with the increase of vapor pressure (Vp) and water solubility (Sw). Specifically, the residual level of PAEs was the highest when Vp was greater than 10^−5^ mmHg and Sw was less than 12 mg/L.

Except for the far-infrared baking stage, the PFs of DBP and DEHP in all the other four stages during processing were higher than 1. A possible reason could be the relatively high Log Kow and Bp, and the relatively low Vp of DBP and DEHP. As a result, DBP and DEHP in the tea samples were less likely to be emitted into the air, and DBP and DEHP in the air and contact substances were adsorbed into the surface of the tea leaves and migrated into the tea leaves more easily. Owing to the extremely low melting point (Mp) of five PAEs, their degradation and dissipation may be due to prolonged high-temperature treatment, and the PF in the finished tea sample was less than 1.

**Figure 6:**
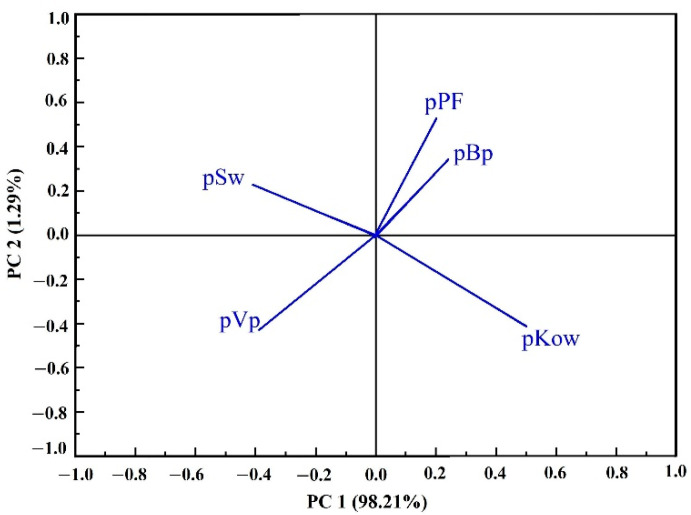
Principal component analysis of processing factors and physicochemical parameters of five PAEs during black tea processing.

Principal component analysis (PCA) of PFs and the physicochemical properties of five PAEs was conducted to determine correlations. As shown in Figure 6, PC1 and PC2 represented 99.50% in the samples, indicating that PCA analysis can reflect the comprehensive information of the samples. The loading values indicate that the levels of five PAE residues during black tea processing mainly depend on the pKow, followed by pSw and pVp. Results indicate that the value of pPF was positively correlated with pKow and pBP, but negatively correlated with pSw and pVp.

## 4. Discussion

In this study, solid-phase extraction was used to enrich and purify PAEs in tea, and then GC–MS was used to qualitatively and quantitatively detect the PAEs in tea. A method was developed to analyze five PAEs in tea, and the extraction conditions were optimized by response surface methodology. In terms of the selection of extraction solvents, n-hexane organic solvent was used because of its weak polarity and strong solubility for PAEs. In addition, the matrix solution extracted with n-hexane has fewer interfering substances compared with other organic solvents [38]. Due to the acid–base instability of PAE structures, sodium chloride was addicted during the pretreatment process to enhance the stability of anthocyanins during the extraction process [39]. Using the optimized conditions, the PAEs in tea can be better extracted, with a high extraction efficiency, high resolution of target chromatographic peaks, and a low LOD of the method. Especially, the method of analyzing five PAEs in tea showed good specificity, reliability, and reproducibility with certain applicability. Compared with the QuEChERS method and the simultaneous distillation extraction method adopted by Yin et al. [23] and Du et al. [28], respectively, this development method extracts PAEs with higher specificity and with a higher extraction rate than the QuEChERS method, and it also avoids the cross-contamination caused by the simultaneous distillation extraction method.

Black tea is a fully fermented tea and is the most-sold tea in the world. Moreover, in the past ten years, China’s black tea series product has developed rapidly, and their output and sales have surged. It is of great practical significance to discuss the inhibitory effect of black tea processing technology on PAEs. The results show that all five PAEs can be detected in each stage of black tea processing, among which DBP accounts for the highest proportion, consisting of 81.5–95.30% of the total amount of PAEs. Except for the finished tea sample, the content of DBP and DEHP in each stage of black tea processing was higher than that in the fresh leaves, which may be because the tea samples were exposed to the air for a long time and contacted equipment and other objects (such as loading machines, conveyors, tea twisting machine, tea drying machinery, and so on), causing PAEs to adhere to the surface of the tea leaves and migrate into the tea. By analyzing their physicochemical properties, we found that the Log Kow and Bp of DBP and DEHP are high, while the Vp and Sw are low, which further explains the enrichment of two PAEs in tea. Some researchers found that the contamination level of polycyclic aromatic hydrocarbons (PAHs) in tea showed an increasing trend during withering and rolling of black tea, which may be caused by environmental factors [40,41], but spreading and drying reduced PAH concentrations [42]. During the far-infrared baking stage, the levels of five PAEs were significantly reduced, indicating that the thermochemical reaction at high temperature may be the main reason for the degradation of PAEs. Liu et al. [29] found that PAEs were significantly reduced during green-tea processing, and considered the drying stage as the key factor for the loss of PAEs.

The PF can effectively indicate the main cause for PAE degradation. The PF value of PAEs after far-infrared baking was less than one during black tea processing, which indicates that the level of PAE residues in dried tea samples is lower than that in fresh leaves. Therefore, the baking stage is the main factor for PAE degradation, and the main reason may be the emission or degradation of PAEs due to prolonged high temperatures. Drying of the tea leaves was carried out in two stages (primary drying and far-infrared baking), and the temperature was high and the time long during the whole process. In addition, the far-infrared mode was adopted in this study; the possibility of thermal volatilization is very high during the high-temperature mode for a long time. Sood et al. [43] also found that water molecules in plant tissue can enter the interior of pesticide molecules, which can promote the volatilization and degradation of pesticides during heating. The PF of PAEs was the highest during the withering process, and then gradually decreased during rolling and fermentation, which may be because of the release of the fresh leaf juice caused by rolling and the enzymes during fermentation. Wang et al. [36] found that the loss of anthraquinone was also associated with rolling and fermentation in the study of the effect of black tea processing on the degradation of anthraquinone. PCA found that the residual level of PAEs in tea was closely related to its Log Kow: the levels of residual PAEs were positively correlated with Log Kow and BP, but negatively correlated with Sw and Vp.

## 5. Conclusions

In summary, our study successfully established a method for the analysis of five phthalate esters in tea using solid-phase extraction coupled with GC–MS. The extraction conditions of five PAE residues in tea were optimized by Box–Behnken response surface design. The final conditions were determined as follows: solid-to-liquid ratio of 1:20, extraction temperature of 40 °C, and extraction time of 12 h. The linearity obtained at *r*^2^ > 0.99 for PAEs ranged from 1 to 1000 μg/L (except for 500 μg/L for DBP). The LODs and LOQs of this method were in the range of 0.2–0.6 μg/kg and 0.7–2.0 μg/kg, respectively. The recoveries of the five PAEs ranged from 80.70% to 98.68%, with RSD 2.7–8.1%. The results showed that the residues of PAEs in each stage of black tea processing mainly depended on Log Kow, followed by Sw and Vp. Prolonged high temperature has a significant effect on the degradation of PAEs. This study quantified the level of PAE residues and investigated the characteristics of PAE residues during black tea processing. The PFs of PAEs during black tea processing can assess the health risk of the residues and evaluate whether it is safe for consumers. This work may serve as a guide for the detection, processing-loss determination, and control of PAE MRLs in tea.

## Figures and Tables

**Table 1:** Design and results of response surface test.

Test Number	Factor			Experimental Content (μg/kg)	Predicted Content (μg/kg)
	Solid–Liquid Ratio (A)	Extraction Temperature (B)	Extraction Time (C)		
1	1:20	40	12	546.14	567.92
2	1:20	40	12	557.63	576.48
3	1:10	50	12	348.29	328.23
4	1:20	40	12	647.50	585.85
5	1:20	40	12	559.27	577.11
6	1:20	40	12	529.08	533.45
7	1:10	40	18	416.98	402.39
8	1:30	30	12	216.75	236.81
9	1:20	30	6	193.37	158.72
10	1:20	50	18	395.63	430.28
11	1:10	30	12	219.02	249.49
12	1:10	40	6	166.01	170.17
13	1:30	50	12	302.30	207.11
14	1:30	40	6	138.57	153.16
15	1:20	30	18	380.81	364.92
16	1:30	40	18	354.47	350.30
17	1:20	50	6	191.22	207.11

**Table 2:** Variance analysis of regression model of PAE extraction from tea.

Source of Variance	Degrees of Freedom	Sum of Square	Mean Square	*F*-Value	*p*-Value	Distinctiveness
A-Solid–liquid ratio	1	2387.40	2387.40	1.15	0.3190	
B-Extraction temperature	1	6468.96	6468.96	3.12	0.1208	
C-Extraction time	1	92,177.15	92,177.15	44.42	0.0003	**
AB	1	477.86	477.86	0.23	0.6459	
AC	1	307.65	307.65	0.15	0.7116	
BC	1	72.00	72.00	0.035	0.8575	
A^2^	1	1.062 × 10^5^	1.062 × 10^5^	51.16	0.0002	**
B^2^	1	79,652.45	79,652.45	38.39	0.0004	**
C^2^	1	82,674.64	82,674.64	39.84	0.0004	**
Model	9	4.018 × 10^5^	44,642.87	21.51	0.0003	**
Residual	7	14,525.52	2075.07			
Lack-of-fit	3	6028.92	2009.64	0.95	0.4980	
Pure error	4	8496.60	2124.15			
Cor total	16	4.163 × 10^5^				

Notes: **, *p* < 0.01 means that the difference is very significant.

**Table 3:** Mass spectrometric information of five PAEs.

Number	Compound	Retention Time (min)	Qualitativeion (*m*/*z*)	Quantitative Ion (*m*/*z*)
1	DMP	12.98	163,77,194,133	163
2	DEP	17.38	149,177,105,222	149
3	DiBP	26.90	149,223,104,167	149
4	DBP	29.50	149,223,205,104	149
5	DEHP	39.57	149,167,279,113	149

**Table 4:** Method validation of linearity, recoveries, limits of detection (LOD) and quantification (LOQ), and matrix effect for five PAEs in tea.

PAEs	Linear Range (μg/L)	*r* ^2^	LOD (μg/kg)	LOQ (μg/kg)	Recoveries, % (RSD, %)			Matrix Effect
					100 μg/L	500 μg/L	1000 μg/L	
DMP	1.00–1000.00	0.9910	0.40	1.33	81.71 (4.32)	95.00 (7.55)	97.07 (5.70)	1.13
DEP	1.00–1000.00	0.9914	0.61	2.03	80.70 (5.60)	94.28 (6.52)	93.88 (4.56)	1.02
DIBP	1.00–1000.00	0.9950	0.42	1.40	82.69 (8.14)	94.22 (4.03)	91.57 (3.31)	0.63
DBP	1.00–500.00	0.9961	0.50	1.66	89.30 (6.67)	90.37 (8.69)	98.28 (4.58)	0.50
DEHP	1.00–1000.00	0.9963	0.21	0.71	84.37 (6.39)	93.99 (3.94)	98.68 (2.72)	0.40

**Table 5:** Content of PAEs (±SD (μg/kg)) in different stages of black tea processing (n = 3).

Sample	DMP	DEP	DIBP	DBP	DEHP	Sum
Fresh leaves sample	12.31 ± 0.39	35.13 ± 0.17	33.15 ± 0.56	430.41 ± 15.08	16.89 ± 1.64	527.90 ± 31.38
Withered sample	11.36 ± 0.35	13.01 ± 0.22	21.73 ± 0.37	1436.37 ± 17.32	24.73 ± 2.29	1507.21 ± 139.04
Rolled sample	11.28 ± 0.87	12.97 ± 0.51	22.68 ± 1.34	1166.90 ± 18.83	26.86 ± 3.99	1240.86 ± 109.02
Fermented sample	11.44 ± 0.39	12.87 ± 0.54	19.13 ± 1.88	895.98 ± 9.16	23.56 ± 1.69	962.97 ± 100.84
First drying sample	11.67 ± 0.40	12.99 ± 0.33	20.77 ± 1.37	1313.84 ± 9.31	28.33 ± 1.52	1387.61 ± 128.37
Finished tea sample	11.26 ± 1.47	12.82 ± 2.68	37.08 ± 3.87	387.69 ± 4.09	11.73 ± 1.72	460.85 ± 4.33

**Table 6:** CAS number, molecular weight (Mw), octanol–water partition coefficient (Log Kow), water solubility (Sw), vapor pressure (Vp), melting point (Mp), boiling point (Bp), and structural formula of five phthalates.

PAEs	CAS Number	Mw	Log Kow	Sw (mg/L, 25 °C)	Vp (mm Hg, 25 °C)	Mp (°C)	Bp (°C)	Structural Formula
DMP	131-11-3	194.19	1.60	4000.00	3.08 × 10^−3^	5.50	282.68	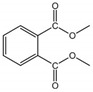
DEP	84-66-2	222.24	2.42	1080.00	2.10 × 10^−3^	−40.50	294.00	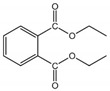
DiBP	84-69-5	278.35	4.11	6.20	4.76 × 10^−5^	−50.00	327.00	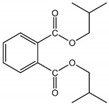
DBP	84-74-2	278.35	4.50	11.20	2.01 × 10^−5^	−35.00	337.00	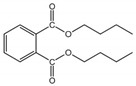
DEHP	117-81-7	390.57	7.60	0.27	1.42 × 10^−7^	−50.00	384.90	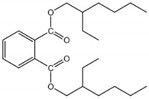

## Data Availability

Data sharing not applicable.

## Supplementary Materials

The following supporting information can be downloaded at: https://www.mdpi.com/article/10.3390/foods11091266/s1, Table S1: Equations for five PAE compounds.
