# Supplementary material for: Determination of Five Phthalate Esters in Tea and Their Dynamic Characteristics during Black Tea Processing
Source: Foods. 2022 Apr 27;11(9):1266. doi: 10.3390/foods11091266 (PMC9103538; doi:10.3390/foods11091266)
Supplement: Supplementary file 1 [file foods-11-01266-s001.zip › foods-1690093-supplementary.pdf]

foods

Supplementary Materials

Determination of Five Phthalate Esters in Tea and Their Dynamic Characteristics during Black Tea Processing

Yanyan Tang †, Mengxin Wang †, Cheng Pan, Shuishan Mi and Baoyu Han *

Zhejiang Provincial Key Laboratory of Biometrology and Inspection and Quarantine, College of Life Sciences, China Jiliang University, Hangzhou 310018, China; yan9268264@163.com (Y.T.); wmx@cjlu.edu.cn (M.W.); pancheng@cjlu.edu.cn (C.P.); mishuishan09@163.com (S.M.)
* Correspondence: hanbaoyu@cjlu.edu.cn; Tel.: +86-135-8808-6214
† These authors contributed equally to this work.

Table S1. Equations for five PAE compounds.

Code	Compound	Equation	Correlation coefficient
1	DMP	y=173757714.7000x- 3839271.0340	0.9910
2	DEP	y=188634775.3000x- 4764391.5410	0.9914
3	DiBP	y=364107998.3000x- 5936135.5340	0.9950
4	DBP	y=456912383.6000x- 3779972.0700	0.9961
5	DEHP	y=198609811.6000x- 1612821.7740	0.9963
